# The L.C.C. and Public Slaughter-Houses

**Published:** 1910-07-30

**Authors:** 


### Editor's Letter-Box.

### THE L.C.C. AND PUBLIC SLAUGHTER-HOUSES.

*To the Editor of* The Hospital.

Sir,—For some years past the Humanitarian League has been urging on the London County Council the need of establishing public slaughter-houses, and has pointed to the fact that this course, advocated in the official reports both of the Royal Commission on Tuberculosis and of the Committee appointed by the Admiralty to consider the Humane Slaughtering of Animals, has also been recommended by a number of leading medical and sanitary authorities, and by the Public Health Committee of the L.C.C. itself.

We have lately received a letter from the Council informing us that it is their opinion that "before any action is taken, an inquiry should be made into the question of the desirability of establishing public slaughter-houses in London," and that the Local Government Board does not see its way at the present time to undertake such an inquiry.

But surely the "desirability," both on humane grounds and hygienic, of substituting public for private slaughter-houses has been already proved beyond question in the official reports and professional testimony to which I have alluded; and it would seem to be a mere waste of time to make yet another inquiry into a matter which has been inquired into again and again.—Yours faithfully,

Secretary Humanitarian League.

53 Chancery Lane, London.

